# Novel FOXM1 inhibitor STL001 sensitizes human cancers to a broad-spectrum of cancer therapies

**DOI:** 10.1038/s41420-024-01929-0

**Published:** 2024-05-02

**Authors:** Sanjeev Raghuwanshi, Xu Zhang, Zarema Arbieva, Irum Khan, Hisham Mohammed, Z. Wang, Alexander Domling, Carlos Jaime Camacho, Andrei L. Gartel

**Affiliations:** 1 University of Illinois at Chicago, Department of Medicine, Chicago, IL USA; 2 Northwestern University, Chicago, IL USA; 3 Oregon Health & Science University, Knight Cancer Institute, School of Medicine, Chicago, IL USA; 4 The Czech Advanced Technology and Research Institute (CATRIN) of Palacký University, Chicago, IL USA; 5 Department of Computational and Systems Biology, University of Pittsburgh, Chicago, IL USA

**Keywords:** Target validation, Oncogenes

## Abstract

Forkhead box protein M1 (FOXM1) is often overexpressed in human cancers and strongly associated with therapy resistance and less good patient survival. The chemotherapy options for patients with the most aggressive types of solid cancers remain very limited because of the acquired drug resistance, making the therapy less effective. NPM1 mutation through the inactivation of FOXM1 via FOXM1 relocalization to the cytoplasm confers more favorable treatment outcomes for AML patients, confirming FOXM1 as a crucial target to overcome drug resistance. Pharmacological inhibition of FOXM1 could be a promising approach to sensitize therapy-resistant cancers. Here, we explore a novel FOXM1 inhibitor STL001, a first-generation modification drug of our previously reported FOXM1 inhibitor STL427944. STL001 preserves the mode of action of the STL427944; however, STL001 is up to 50 times more efficient in reducing FOXM1 activity in a variety of solid cancers. The most conventional cancer therapies studied here induce FOXM1 overexpression in solid cancers. The therapy-induced FOXM1 overexpression may explain the failure or reduced efficacy of these drugs in cancer patients. Interestingly, STL001 increased the sensitivity of cancer cells to conventional cancer therapies by suppressing both the high-endogenous and drug-induced FOXM1. Notably, STL001 does not provide further sensitization to FOXM1-KD cancer cells, suggesting that the sensitization effect is conveyed specifically through FOXM1 suppression. RNA-seq and gene set enrichment studies revealed prominent suppression of FOXM1-dependent pathways and gene ontologies. Also, gene regulation by STL001 showed extensive overlap with FOXM1-KD, suggesting a high selectivity of STL001 toward the FOXM1 regulatory network. A completely new activity of FOXM1, mediated through steroid/cholesterol biosynthetic process and protein secretion in cancer cells was also detected. Collectively, STL001 offers intriguing translational opportunities as combination therapies targeting FOXM1 activity in a variety of human cancers driven by FOXM1.

## Introduction

Forkhead box (FOX) protein M1 (FOXM1) is a transcription factor (TF) in the forkhead box superfamily of TFs, characterized by a conservative DNA-binding domain (DBD) [1, 2]. As a proliferation-specific TF, FOXM1 is implicated in the regulation of several cellular processes such as cell cycle progression, cell division, DNA damage repair, metabolism, angiogenesis, redox signaling, inflammation, and apoptosis [3, 4]. FOXM1 is abnormally overexpressed and amplified in the majority of human cancers (such as ovarian cancer, colorectal cancer, esophageal cancer, breast cancer, prostate cancer, gastric cancer, and pancreatic cancer) [5–8] and well demonstrated as a master transcriptional regulator in cancer development [9, 10]. Indeed FOXM1 has emerged as a key oncogenic driver of cell division, aggressiveness, metastasis, and drug resistance of cancer cells [5, 6, 9–12]. High FOXM1 levels are generally associated with therapeutic resistance of cancer cells and poor prognosis of cancer patients due to decreased efficacy of the traditionally used therapeutic strategies [1–3, 5, 13, 14], it shows that FOXM1 may serve as a selective target in human solid cancers.

Clinically, chemotherapy is the major therapeutic approach for the conventional and essential treatment of cancer patients [3]. Several anti-cancer medicines are being used in the clinic as the primary support of the current treatment to reduce the death of cancer patients [15]. However, inherent or acquired resistance to chemotherapeutic drugs (e.g., 5-FU, Cisplatin, Paclitaxel and Carboplatin, Doxorubicin, and Tamoxifen) remains the major contributor to therapy failure in solid cancers [15–18]. The underlying mechanisms governing the development of chemoresistance are complicated and the leading pathway(s) are ill-defined. While multiple mechanisms can mediate drug resistance development, FOXM1 is repeatedly identified as a common factor associated with weaker responses to conventional cancer therapies by regulating several target genes associated with cell cycle and DNA repair [3, 5, 18–24]. There are diverse molecular mechanisms of cancer drug resistance mediated through FOXM1, including enhanced DNA damage repair [23–25], oxidative stress prevention [26–28], increased drug efflux activity [20, 29–31], increased thymidylate synthase (TS) activity [32, 33], the negative regulation of the JNK/mitochondrial pathway [34], induction of AMPK/mTOR signaling [21], or via up-regulating microtubule dynamics regulation associated components and blocking drug-induced mitotic catastrophe [35, 36]. Accordingly, the knockdown of FOXM1 or its downstream targets was found very effective in restoring standard chemotherapy sensitivity in many human cancers, including colorectal cancer [20, 32], Lung carcinoma [34], breast cancer [36], ovarian cancer [19], prostate cancer [21], esophageal cancer [8]. Moreover, we have recently reported that the FOXM1 is transcriptionally downregulated in NPM1^mut^ AML patients and it is an independent predictor of drug response [37]. Therefore, FOXM1 inhibition in combination with chemotherapy may prove critical to overcome drug resistance in solid cancer patients.

**Fig. 1:**
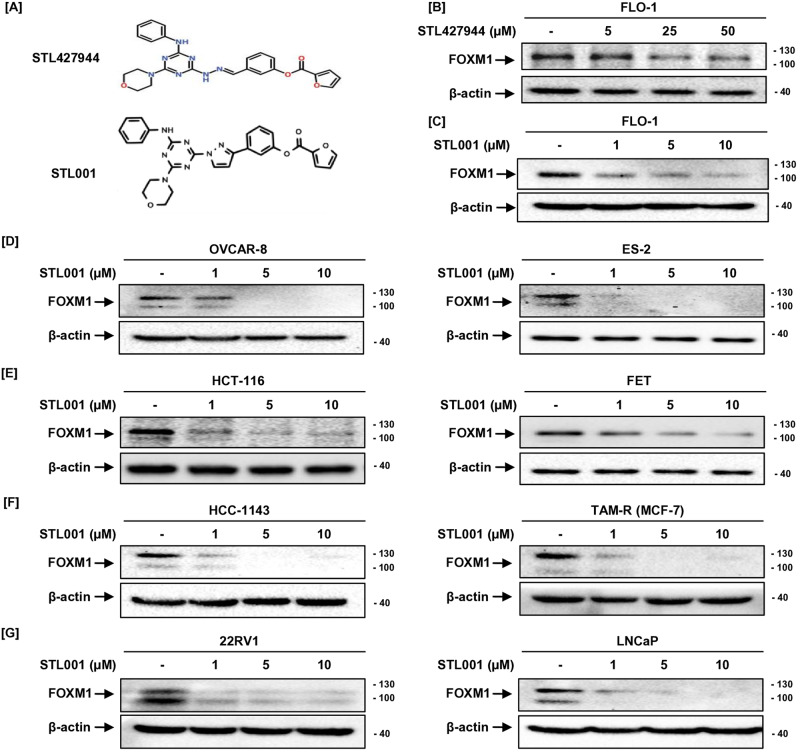
STL001 causes dose-dependent suppression of FOXM1 protein levels in a variety of solid tumor-derived cancer cell lines. **A** The structural formula of novel FOXM1 inhibitor STL001 and its precursor molecule STL427944, modified from source [5, 37]. **B**–**G** Various cancer cell lines were treated with increasing concentrations of STL427944 (**B**) and STL001 (**C**–**G**) for 24 h. Total protein samples obtained from treated cells were analyzed for FOXM1 protein levels via immunoblotting, and β-actin was used as internal loading control (*n* = 3 for each group). The STL001 was 25–50 times more efficient in reducing cellular FOXM1 protein levels in a variety of solid cancers as compared to its parental compound STL427944.

The lead role of FOXM1 in cancer development and chemoresistance prompted new developments in the research field of FOXM1 small-molecule inhibitors (SMIs). Pharmacological inhibition of FOXM1 by SMIs is a promising approach to overcome chemoresistance, in the recent past, several FOXM1 inhibitors have been established, but they have various mechanisms with less defined efficacy and selectivity [1, 38], therefore FOXM1 targeting is a challenging task in pursuit of clinical translatability. However, in the present time, the development of high-quality FOXM1 inhibitors is an important health need. We have previously described the compound STL427944, a first-in-class SMI of FOXM1 [5], that was identified by network-centric transcriptomic analysis and confirmed as a selective inhibitor of FOXM1. STL427944 was found to block FOXM1 activity by inducing the relocalization of nuclear FOXM1 to the cytoplasm and promoting its autophagic degradation, it sensitizes various cancer cells to multiple traditionally used chemotherapies at very high concentrations [5]. Though possessing high selectivity toward FOXM1, the compound STL427944 has metabolic liabilities, to overcome these issues, we applied several paths of Structure-Activity Relationship (SAR) optimization of selected active compounds; among ten newly designed analogues, STL001 showed up to a 50-fold estimated increase in potency as FOXM1 inhibitor (Fig. 1A). The novel compound, STL001 was studied further to verify its direct target engagement with FOXM1, resulting in at least 10-fold more active compound that preserved the mode of action of its parental compound, STL427944 [37]. Both the compounds have shown similar activity in AML, however, STL001 has greater drug-like properties and thus enhanced potency [37].

Therefore, we sought to investigate the anticancer effects of the novel FOXM1 inhibitor, STL001 in a variety of human cancers from solid tumors including ovarian, colorectal, breast, esophageal, and prostate cancers. Here, the capacity of STL001 as a FOXM1 inhibitor was verified in human cancer cell lines from solid tumors. STL001 was studied further to verify its direct target engagement with FOXM1. We have also provided Transcriptome-supported evidence that STL001 exhibits selectivity toward suppressing FOXM1-controlled regulatory pathways. This study verifies and characterizes a novel FOXM1 inhibitor STL001 that effectively antagonizes FOXM1 activity and sensitizes a variety of human cancers to a broad spectrum of traditionally used chemotherapy agents and may be suitable for further clinical evaluation in targeting chemotherapy-resistance solid tumors.

## Results

### STL001 decreased FOXM1 protein expression levels in human cancer cells of different etiology

We have previously described a first-in-class small molecule inhibitor of FOXM1, STL427944. The STL427944 was identified by transcriptomic network analysis, which is a network-centric strategy and focuses on the pathways that mediate both biology and pathophysiology [5]. The conventional target-centric drug discovery strategies prioritize single-target potency and molecules that directly interact with a protein of interest. The requirement for direct binding without considering connectivity and multi-target effects within a pathway network significantly limits the number of options and increases the chances of identifying agents with unwanted nonspecific effects. As an alternative to target-centric drug development, we applied this new network-centric screening concept and identified the compound STL427944 using differentially expressed gene signatures from the LINCS L1000 dataset [5]. The STL427944 reduces the chemoresistance of cancer cells by inducing FOXM1 degradation and is confirmed in various cancer cell lines as a selective inhibitor of the FOXM1 pathway at very high concentrations [5]. However, from a medicinal chemistry perspective, the compound STL427944 has metabolic liabilities, to overcome these issues; we did structural modifications by applying several paths of SAR optimization and verified the potency, among the newly designed STL427944 analogues, STL001 (Fig. 1A) showed up to a 50-fold estimated increase in potency as FOXM1 inhibitor in AML [37]. The novel FOXM1 inhibitor STL001 is a new molecule with similar biological properties to the parent compound STL427944, however, the ring replacement in the parental compound is likely to have significantly improved the overall stability and better drug-like properties and thus enhanced potency observed in its derivative, STL001 [37].

To assess experimentally the FOXM1-suppressing effect of the STL001 in solid cancer, we used a panel of human solid tumor-derived cell lines with high FOXM1 expression levels, including ovarian cancer (OVCAR-8, ES-2), colorectal cancer (HCT-116, HCT-FET), esophageal cancer (FLO-1), hormone receptor-positive (TAM-R) and triple negative (HCC-1143) breast cancers, and prostate cancer (22Rv1, LNCaP). All cell lines were treated with STL001 (1, 5, and 10 μM) resulting in a dose-dependent reduction of the cellular levels of FOXM1 protein at significantly lower concentrations (1 μM, Fig. 1C–G) when compared with its precursor (STL427944) that shows modest FOXM1 suppression at concentrations of 25–50 μM (Fig. 1B, Supplementary Fig. 1A). These results demonstrate that the STL001 is a universal inhibitor of FOXM1 in cancer cells, also it is 25–50 times more efficient in reducing the cellular FOXM1 activity in solid cancer as compared to its parental compound STL427944 [5].

### STL001 induces the translocation of nuclear FOXM1 to the cytoplasm and promotes its autophagic degradation

**Fig. 2:**
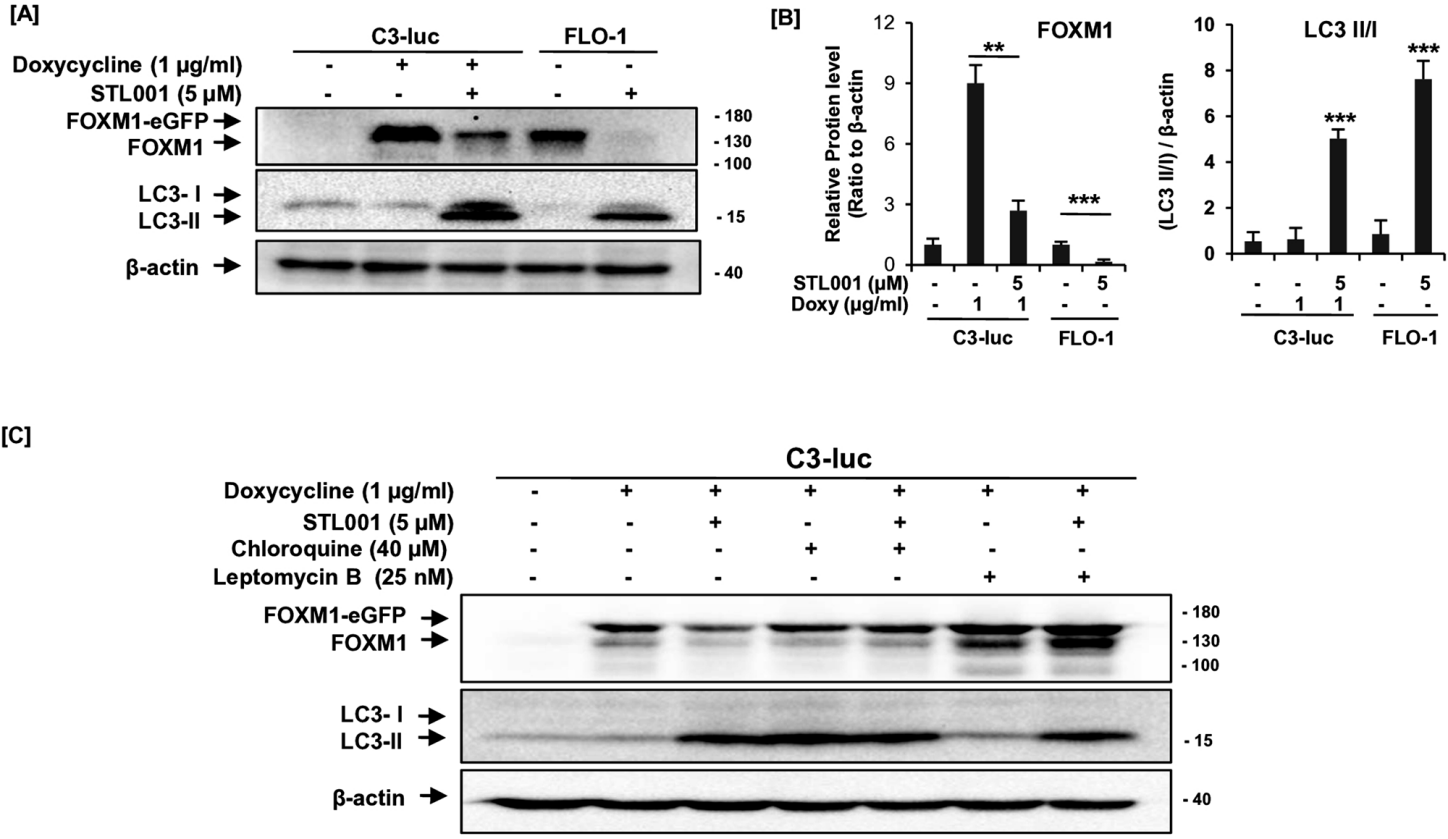
FOXM1 suppression by STL001 is the autophagy-mediated process and is prevented by nuclear-export arrest. **A** U2OS-C3-luc cells expressing doxycycline (Doxy)-inducible EGFP-FOXM1 fusion protein and FLO-1 cells were treated with STL001 for 24 h. Total protein samples were analyzed via immunoblotting for FOXM1 and LC3 expression, and β-actin was used as an internal loading control. **B** The bar graph represents the differential expression of FOXM1 and the ratio of LC3 II/I, analyzed by blot densitometry and presented as means ± S.D from three independent experiments (*n* = 3; **p < 0.001, ***p < 0.0001 using two-tailed Student’s *t*-tests). **C** U2OS-C3-luc cells were treated with indicated concentrations of doxycycline, STL001, chloroquine, and leptomycin B for 24 h. Total protein samples obtained from treated cells were analyzed for FOXM1 and LC3 expression levels via immunoblotting, and β-actin was used as an internal loading control (*n* = 3 for each group).

The parental compound, STL427944 affects FOXM1 activity via a two-step mechanism; first, it induces the translocation of nuclear FOXM1 to the cytoplasm, followed by autophagosomal degradation of FOXM1 protein [5]. In the present study, we have also verified whether the mechanism of STL001-mediated FOXM1 suppression is similar to its parental compound STL427944. To verify the induction of autophagy by STL001, we examined the autophagy marker protein, LC3- II/I. Indeed, treatment of U2OS-C3-luc cells that express doxycycline (Doxy)-inducible EGFP-FOXM1 fusion protein [39] and FLO-1 cells with STL001 for 24 h resulted in reduced FOXM1 levels with increased expression of autophagy marker protein LC3 (Fig. 2A, B). Further, we have tested this mechanism by using chloroquine, a well-known drug that prevents autophagosome-lysosome fusion [5]. The addition of chloroquine did not affect FOXM1 protein levels in C3-luc cells; however, it completely rescued FOXM1 protein from suppression by STL001 (Fig. 2C). It shows that the autophagosome-lysosome fusion and maturation into autolysosomes is essential for STL001 effect on FOXM1. In the process of autophagic degradation, the nuclear FOXM1 is translocated to the cytoplasm [5]. To understand the functional role of STL001 in the translocation of nuclear FOXM1, we arrested the nuclear-protein export with leptomycin B, resulting in a complete reversal of STL001-induced FOXM1 suppression without any effect on autophagy activation (Fig. 2C). This data suggest that the translocation of nuclear FOXM1 to the cytoplasm is a crucial event to make FOXM1 available to autophagosomes. These results demonstrate that the first-generation modification drug, STL001, preserves the mode of action of the parent compound, STL427944, however, the exact mechanisms of FOXM1 translocation to the cytoplasm and the induction of autophagy would require detailed investigation in the future.

### RNA-seq analysis of the effects of STL001 and of FOXM1-KD on global FOXM1 regulatory network

**Fig. 3:**
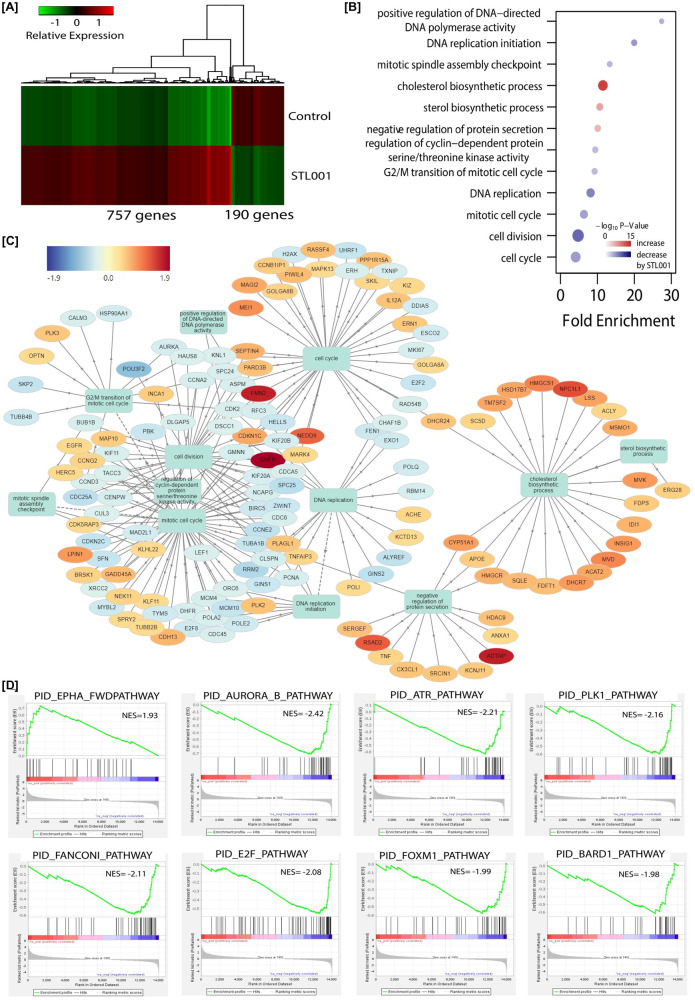
RNA-Seq analysis of the gene expression by STL001 treatment in esophageal cancer cells. **A** Heatmap of mean gene expression levels in control and STL001 (5 µM) for 947 differentially expressed genes (DEG). **B** Gene enrichment analysis of the 947-DEG in Gene Ontology biological processes. The size of points is proportional to the number of overlapped differential genes in the biological processes. **C** The network output of the most enriched biological processes and associated proteins was generated by the GOnet tool. Arrows indicate a direct link between most enriched biological processes according to the GOnet annotation. Round nodes represent proteins; square nodes represent enriched biological processes. **D** Gene set enrichment analysis of differential gene expression using Pathway Interaction Database (PID) collection of gene signature [PMID: 18832364].

**Fig. 4:**
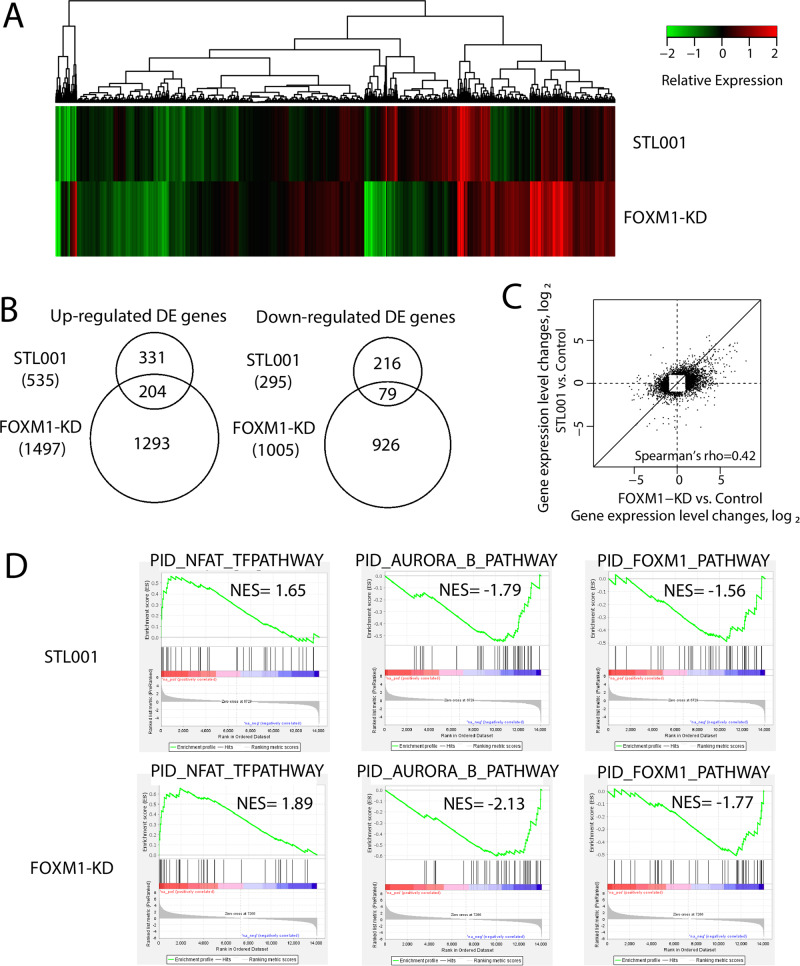
Differential gene expression shared between STL001 treatment and FOXM1-KD in ovarian cancer. **A** Heatmap of mean gene expression changes relative to Control in STL001 treatment and FOXM1-KD for 14,980 analyzed genes. **B** Venn diagrams of up-and down-regulated genes in STL001 treatment and FOXM1-KD. **C** Scatter plot of log2 gene expression changes in STL001 treatment and FOXM1-KD, for 3018 genes differentially expressed in either STL001 treatment or FOXM1-KD. **D** Gene set enrichment analysis of differential gene expression using Pathway Interaction Database (PID) collection of gene signature [PMID: 18832364]. Presented are pathways significant at FDR < 0.1 in one condition and with nominal *P*-value < 0.01 in the other condition.

STL001 is a novel compound, its biological activities and molecular targets are not characterized yet. To investigate the effects of this compound on gene regulation more globally, we used RNA-Seq. We did full transcriptome RNA-seq and analyzed the patterns of gene expression by STL001 in esophageal cancer (FLO-1) cells (Fig. 3) and differential gene expression shared between STL001 treatment and FOXM1-KD in ovarian cancer (OVCAR-8) cells (Fig. 4). Processed data on differential gene expression by STL001 or FOXM1-KD are available in Supplementary Tables 2, 3 and 4).

In this study, out of 16,275 protein-coding genes evaluated, we identified a set of 947 genes showing highly significant (2-fold or more) differential expression (DE) in the STL001 treated experimental model, with 757 genes being upregulated and 190 genes being repressed in FLO-1 (Fig. 3A). We therefore considered the genes displaying expression changes in FLO-1 as the most reliable STL001 responders, we pooled them into the “STL001 signature” gene list (947 DE genes), and subjected them to further analysis. We performed Gene Ontology enrichment analysis of the 947 DE genes, in the category of biological process (Fig. 3B). The purpose of this study was to determine whether the DE genes had significant enrichment trends in some functional types. Functional classification found a total of 12 significantly enriched processes, nine categories were significantly decreased and three were significantly increased by STL001 (Fig. 3B). Notably, the mitotic cell division and DNA replication processes were mainly decreased by STL001 (Fig. 3B), suggesting that STL001 mainly affected the FOXM1-targeted gene network. In addition, cluster analysis was performed on DE genes (947) and 10 significantly enriched biological processes using the GOnet tool. This analysis highlighted clearly linked functional clusters under FOXM1 regulation, such as cell cycle, mitotic cell division, spindle assembly, and DNA replication (Fig. 3C). One cluster related to steroid/cholesterol biosynthetic process and negative regulation of protein secretion was also linked with the core biological functions affected and highlighted with all the upregulated genes (Fig. 3C).

Further, to verify STL001 selectivity toward FOXM1 regulatory pathways, Gene-Set Enrichment Analysis (GSEA) was performed for “STL001 signature” genes (947 DE genes) using the canonical pathway gene signatures database, PID. Out of 947 signatures analyzed, 8 gene sets were significantly enriched in FLO-1 cells treated with STL001 (Fig. 3D). Out of 8 gene sets, 7 displayed negative normalized enrichment scores (Fig. 3D), predicting inactivation of these pathways by STL001. It is noteworthy that AURKB and PLK1 are the well-known targets of FOXM1 through direct interaction with their promoters [40], while ATR, BARD1, and FANCONI are DNA damage response (DDR) pathways, several crucial gene components of these pathways are regulated by FOXM1 in DNA damage repair [10]. Moreover, E2F pathway activity can be affected by FOXM1 expression [41], implying that the pathways affected by STL001 converge to the FOXM1-regulated protein network that is involved in tumor survival and resistance to drugs. Taken together, this data shows a very high probability of FOXM1 being the main mediator of STL001 effects on gene expression program in esophageal cancer cells.

To further confirm the selectivity of STL001 toward FOXM1 regulatory pathways we analyzed the differential gene expression shared between STL001 treatment and FOXM1-KD in ovarian cancer (OVCAR-8) cells via full Transcriptome RNA-seq. Figure 4A shows the hierarchical clustering of the differentially expressed genes (DEGs). In Fig. 4B, the differences and overlaps of genes in the STL001 and FOXM1-KD groups are shown using a Venn diagram. Out of 16275 protein-coding genes evaluated, we identified a set of 830 and 2502 genes showing highly significant (2-fold or more) differential expression (DE) in the STL001 and the FOXM1-KD experimental model, respectively (Fig. 4B). In the STL001 group, 535 genes are upregulated and 295 genes are repressed in OVCAR-8 cells, whereas in the FOXM1-KD group, 1497 and 1005 genes are up- and down-regulated, respectively (Fig. 4B). We found that 204 up-regulated DE-genes (~62%) and 79 down-regulated DE-genes (~37%) by STL001 overlapped with the FOXM1-KD group (Fig. 4B), indicating that STL001 affects the FOXM1 gene network. The Fig. 4C scatter plot of log2 gene expression changes in STL001 treatment and FOXM1-KD shows a correlation between DE genes by STL001 treatment and FOXM1-KD (Spearman’s rho = 0.42). This overlap of transcriptomic effects caused by either STL001 or FOXM1-KD confirms the idea that the effects of STL001 are through suppression of FOXM1 activity.

Further, Gene-Set Enrichment Analysis (GSEA) was performed for “STL001 signature” genes (830 DE genes) and 2502 DE genes in FOXM1-KD using the canonical pathway gene signatures database, PID. In GSEA analysis of DE genes of STL001 or FOXM1-KD group, 3 gene sets were significantly enriched in both the groups (Fig. 4D). Two gene sets (PID_AURORA_B_Pathway and PID_FOXM1_Pathway) displayed negative normalized enrichment scores (Fig. 4D), predicting the inactivation of these pathways by STL001 or FOXM1-KD. However, only one gene set (PID_NFAT_TF pathway) displayed a positive normalized enrichment score. The FOXM1_Pathway in PID (Fig. 4D) is a predefined collection of the FOXM1 transcription factor network that is involved in cell cycle regulation and DNA damage repair, and it promotes tumor cell proliferation. A total of 40 genes from 7 different gene families are engaged in this pathway, including tumor suppressors, the oncogenes, genes encoding cyclins and cyclin-dependent kinases, different transcription factors, and protein kinases, e.g., such as PLK1 and AURKB, as well as FOXM1 itself. FOXM1 pathway is the top enriched pathway in many human cancers [5, 42]. It is noteworthy that PID_AURORA_B_Pathway (Fig. 4D) which is involved in the proliferation of cancer cells by positive regulation of cell cycle and G2/M phase transition also represents the activity of direct FOXM1 downstream effectors [5, 40]. Moreover, some of the stress response genes involved in the PID_NFAT_TF pathway (Fig. 4D) can be affected by FOXM1 expression, implying that the pathways affected by STL001 or FOXM1-KD converge to the FOXM1-regulated protein network. Moreover, DE genes of cholesterol biosynthetic pathways affected by STL001 in both the FLO-1 and OVCAR-8 exhibited a prominent positive correlation with FOXM1-KD (Table 1). Taken together, this data shows a very high probability of FOXM1 being the main mediator of STL001 effects on gene expression program in cancer cells.

**Table 1:** Differential gene expression of cholesterol biosynthetic pathways shared between STL001 treatments and FOXM1-KD.

SN.	Gene Name	Gene expression changes log2 fold change (*p* < 0.05)		
		FLO-1_STL001	OVCAR-8_STL001	OVCAR-8 (FOXM1_KD)
1.	DHCR24	1.526395424	1.426395424	1.726395424
2.	MSMO1	1.953436883	1.866905302	1.796905302
3.	SC5D	1.213421737	1.313421737	3.776874947
4.	DHCR7	2.277264256	1.253611991	1.323611991
5.	RSAD1/2	3.299360696	1.053616972	4.006732744
6.	NPC1L1	3.529258581	4.675371408	6.705199651
7.	INSIG1	2.275638578	2.345350987	1.037036562
8.	TNFAIP3	1.150872916	1.069915256	2.987135848
9.	APOE	1.344291161	1.544291161	1.408651275
10.	HDAC9	1.584113601	1.736680718	2.347779157

### STL001-induced FOXM1 suppression sensitizes human cancers of different origin to a broad-spectrum of cancer therapies

Chemoresistance is a major barrier for the traditionally used anti-cancer drugs, while FOXM1 overexpression is closely associated with chemoresistance [3, 13, 14, 38] and poor survival in most solid tumors [6, 9]; whereas, FOXM1 down-regulation is proven very effective in restoring chemotherapy sensitivity in several human cancers [5, 37, 38]. Considering FOXM1 as a critical regulator of sensitivity and resistance in human cancers, we assumed that STL001 treatment-induced FOXM1 suppression should reduce chemoresistance and sensitize human cancer cells to the cytotoxic effects of the relevant cancer chemotherapies. In the present study, we have explored the sensitization effects of STL001 in combination with a broad spectrum of relevant anticancer drugs with different mechanisms of action: direct DNA damage (cisplatin, doxorubicin, and irinotecan), DNA synthesis inhibition (5-FU), mitosis disruption (paclitaxel), and a selective estrogen receptor modulator (tamoxifen) in a verity of human solid cancer-derived cell lines of different etiology.

In this scenario, initially, we have tested if STL001 can sensitize esophageal cancer cells to traditionally used anticancer treatment options [43]. Esophageal cancer is a highly aggressive malignancy of the gastrointestinal tract with 5-year patient survival ranging from 10 to 20% depending on molecular characteristics. Due to high mutational frequency and high ability of invasion, esophageal cancer ranks 7th in incidence and the 6th leading cause of cancer-related mortality worldwide [44]. Recently, over-expression of FOXM1 has been associated with malignant progression of esophageal cancer [7]. Cisplatin, irinotecan, 5-FU, and paclitaxel are traditionally used in esophageal cancer treatment. However, resistance of esophageal cancer to chemotherapeutic agents, e.g., 5-fluorouracil, cisplatin, and paclitaxel, is a major challenge to successfully treat this malignancy [44].

**Fig. 5:**
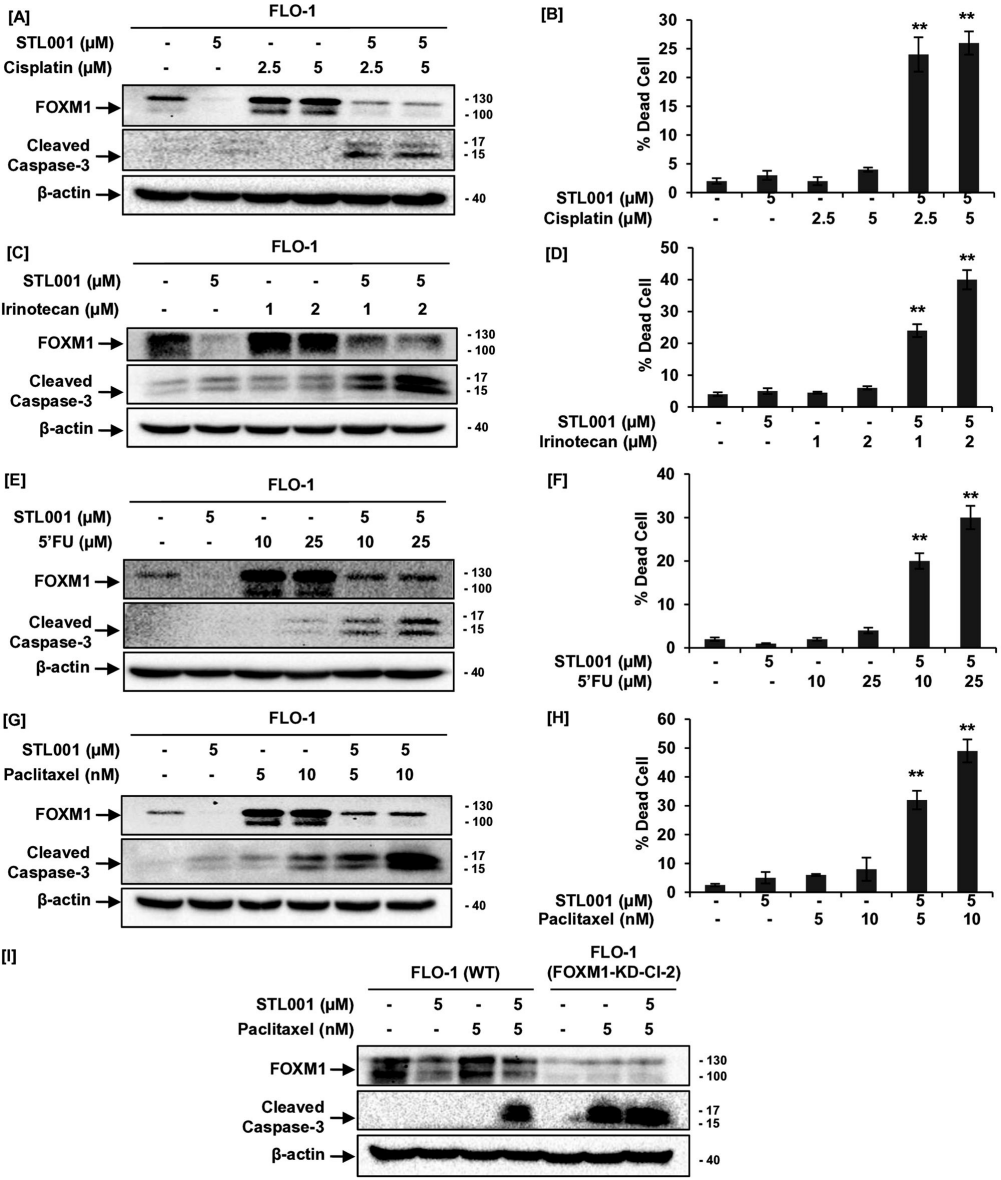
STL001-mediated FOXM1 suppression sensitizes esophageal cancer (FLO-1) cells to a broad spectrum of chemotherapeutic agents with different mechanisms of action. **A**, **C**, **E**, **G** FLO-1 cells were treated with indicated concentrations of cisplatin, irinotecan, 5’FU, paclitaxel, and STL001 alone or in combination with STL001 for 24 h. In all cases, total protein samples were obtained from cells immediately after treatment and analyzed for FOXM1, cleaved caspase-3 levels via immunoblotting, and β-actin was used as internal loading control (*n* = 3 for each group). **B**, **D**, **F**, **H** Percent (%) dead cells in FLO-1 cells treated with indicated concentrations of cisplatin, irinotecan, 5’FU, paclitaxel, and STL001 alone or in combination with STL001 for 24 h. The results shown are the mean ± SEM of three independent experiments performed in triplicate (**p < 0.001 vs control, using two-tailed Student’s *t*-tests; *n* = 3). **I** FLO-1 cells with stable shRNA-mediated FOXM1-KD were treated with paclitaxel alone or in combination with STL001 for 24 h and compared to parental cells under the same treatment conditions. In all cases, total protein samples were obtained from cells immediately after treatment and analyzed for FOXM1 and cleaved caspase-3 levels via immunoblotting, and β-actin was used as internal loading control (*n* = 3 for each group).

In the present study, esophageal cancer (FLO-1) cells treated with different anticancer drugs such as platinum-based DNA damaging agents (cisplatin), the DNA topoisomerase I inhibitors class of drugs (irinotecan) [45], and 5-FU that interferes with thymidine nucleotide synthesis [46]. Cisplatin, irinotecan, or 5-FU at sublethal concentrations significantly increased cellular FOXM1 protein abundance (Fig. 5A, C, E). However, the addition of STL001 in combination with cisplatin, irinotecan, or 5-FU efficiently prevented drug-induced FOXM1 activation, resulting in reduced FOXM1 protein levels when compared with corresponding controls (Fig. 5A, C, E). Notably, as a single agent, STL001 did not exert significant cytotoxic effects (Supplementary Fig. 1B, C), but cells treated with chemotherapy in combination with STL001 led to potent induction of apoptotic cell death (indicated by caspase-3 cleavage) when compared with cells treated with chemotherapy alone (Fig. 5A, C, E). These results indicate that the suppression of FOXM1 activity by STL001 can sensitize esophageal cancer cells to direct or indirect DNA damage-inducing therapies.

Taxanes (Paclitaxel or Docetaxel) are another class of antitumor drugs that have been traditionally used in esophageal cancer treatment [43, 44]. However, chemoresistance associated with high FOXM1 levels in cancer cells decreased the efficacy of these therapies against cancer cells [5, 44]. However, unlike DNA-damaging agents (eg, cisplatin, irinotecan, or 5-FU), taxanes are known to modify mitotic spindle microtubule depolymerization dynamics instead of disrupting cell division [47]. In line with our previous results, esophageal cancer cells treated with paclitaxel (Taxol) at sublethal concentrations showed significantly higher levels of cellular FOXM1 protein without showing prominent cytotoxic effects (Fig. 5G). However, STL001 in combination with taxol enhances the cytotoxic effects of taxol-chemotherapy, detected by induction of strong apoptotic cell death indicated by caspase-3 cleavage (Fig. 5G). These results demonstrate that the chemosensitization effect of STL001 is not limited to DDR regulation and can be much more universal.

Caspases are the primary drivers of apoptotic cell death and caspase-3 cleavage and activation is a common event in apoptotic cell death. While caspases-3 is well-known to play a central role in apoptosis, we sought to verify cell death using a different method for better reliability. Therefore, Trypan blue dye exclusion assay with direct counting was used to assess the cytotoxic effects of STL001 in combination with other drugs (Fig. 5B, D, F, H). In strong agreement with trends observed using the immunoblotting approach, Fig. 5B, D, F, and H illustrate the significant increase in cell mortality in FLO-1 cells after exposure to STL001 in combination with other drugs for 24 h. These results confirmed that STL001 sensitizes esophageal cancer cells to cytotoxic effects of a broad spectrum of the relevant esophageal cancer therapies.

Further, we assess whether this compound sensitizes esophageal cancer cells to different chemotherapeutic drugs via mechanisms besides FOXM1 suppression. To test this, we generated FLO-1 cells with stable shRNA-mediated FOXM1-knockdown (FOXM1-KD). As expected, FOXM1-deficient FLO-1 cells showed increased sensitivity to taxol (Fig. 5I), detected by potent induction of apoptosis indicated by caspase-3 cleavage (Fig. 5I); however, the sensitization effect of STL001 was absent in FLO-1 cells with stable FOXM1-KD (Fig. 5I). These findings suggest that FOXM1 is a crucial factor in esophageal cancer chemoresistance and mediates the effects of STL001 in the sensitization of esophageal cancer to different chemotherapies.

Considering these findings, we assumed that STL001-induced FOXM1 suppression should reduce chemoresistance in any of the different etiology of cancer cells. In this scenario, finally, we have tested the sensitization effect of STL001 to anticancer drugs in model cell lines belonging to solid tumors (such as colorectal cancer, ovarian cancer, breast cancer, and prostate cancer) of different etiology.

**Fig. 6:**
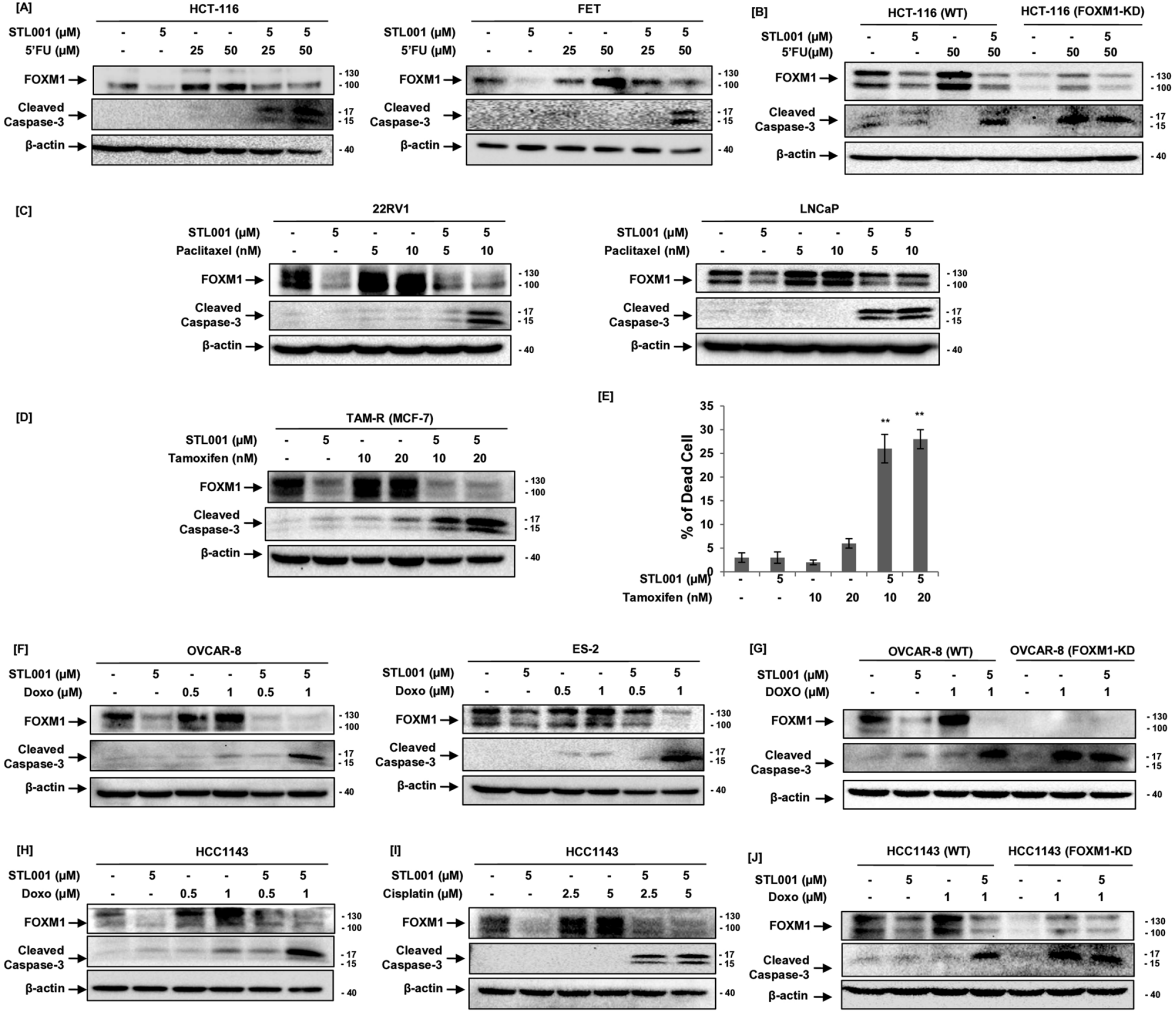
STL001 enhances the cytotoxic effect of conventional chemotherapies through suppression of FOXM1 in a variety of solid cancers. **A** Colon cancer (HCT-116 and FET) cells were treated with indicated concentrations of 5-FU and STL001 alone or in combination for 24 h. **B** HCT-116 cells with stable shRNA-mediated FOXM1-KD were treated with 5-FU alone or in combination with STL001 for 24 h and compared to parental cells under the same treatment conditions. **C** Prostate cancer (22RV1 and LNCaP) cells were treated with indicated concentrations of paclitaxel and STL001 alone or in combination for 24 h. **D** Tamoxifen resistance MCF-7 breast cancer cells (TAM-R) were treated with indicated concentrations of tamoxifen and STL001 alone or in combination for 24 h. In all cases, total protein samples were obtained from cells immediately after treatment and analyzed for FOXM1 and cleaved caspase-3 levels via immunoblotting, β-actin was used as internal loading control (*n* = 3 for each group). **E** Percent (%) dead cells in TAM-R cells treated with indicated concentrations of tamoxifen alone or in combination with STL001 for 24 h. The results shown are the mean ± SEM of three independent experiments performed in triplicate (**p < 0.001 vs control, using two-tailed Student’s *t*-tests; *n* = 3). **F** Ovarian cancer (OVCAR-8 and ES-2) cells were treated with indicated concentrations of doxorubicin (Doxo) and STL001 alone or in combination for 24 h. **G** OVCAR-8 cells with stable shRNA-mediated FOXM1-KD were treated with doxorubicin alone or in combination with STL001 for 24 h and compared to parental cells under the same treatment conditions. **H**, **I** Triple-negative breast cancer cells (HCC1143) were treated with indicated concentrations of doxorubicin (Doxo) (**H**) and cisplatin (**I**) alone or in combination with STL001 for 24 h. **J** HCC1143 cells with stable shRNA-mediated FOXM1-KD were treated with doxorubicin alone or in combination with STL001 for 24 h and compared to parental cells under the same treatment conditions. In all cases, total protein samples were obtained from cells immediately after treatment and analyzed for FOXM1, cleaved caspase-3 levels via immunoblotting, and β-actin was used as internal loading control (*n* = 3).

Colorectal cancer is among the most lethal and prevalent malignant tumors worldwide. The colorectal cancer meta-analysis associates high FOXM1 expression with a poor 5-year survival of patients [48]. 5-FU is one of the most frequently used chemotherapy for the treatment of solid cancers. Also, it is the main first-line chemotherapy used for colorectal cancers; however, resistance to 5-FU therapy exists, resulting in a low 5‐year survival rate [32, 49]. Similar to esophageal cancer, STL001 in combination with 5-FU therapy remarkably decreased 5-FU-induced FOXM1 levels and significantly enhanced the sensitivity of colorectal cancer cells (HCT-116 and FET) to the cytotoxic effects of 5-FU therapy (Fig. 6A). However, the sensitization effect of STL001 was absent in HCT-116 cells with stable shRNA mediated FOXM1-KD (Fig. 6B). Further, we have tested the sensitization effect of STL001 in prostate cancer that is the most commonly diagnosed cancer and the second leading cause of cancer death in males. FOXM1 TF is highly expressed in prostate cancer cells and contributes to cancer development and taxanes (paclitaxel or docetaxel) resistance [50]. Taxanes are a different class of chemotherapy drugs that affect microtubule dynamics during cell division and are widely used to treat a variety of human cancers [51]. However, tumor cells develop resistance to paclitaxel (Taxol), restricting its application for the treatment of cancer patients [52]. In line with our previous results in esophageal cancer cells, the treatment of prostate cancer cells with STL001 in combination with taxol enhanced the cytotoxic effects of taxol therapy, detected by induction of strong apoptotic cell death indicated by caspase-3 cleavage (Fig. 6C). This data further confirms that FOXM1 is a universal factor involved in therapeutic resistance in cancer cells.

Breast cancer is now the most commonly diagnosed cancer in women, about 80% of all breast cancers are positive for estrogen receptors (ER+). FOXM1 is highly expressed in different types of breast cancer and is associated with poor prognosis and chemotherapy resistance [3]. Currently, endocrine therapy is a major treatment option for ER+ breast cancer. Tamoxifen is a selective estrogen receptor modulator (SERM), and it is commonly used to treat all stages of hormone-dependent or ER+ breast cancers, however, the efficacy of tamoxifen as a breast cancer therapy is not satisfactory because of the development of resistance to tamoxifen [53]. In this context, we determine the role of FOXM1 in tamoxifen resistance, in the present work, tamoxifen treatment of TAM-R cells resulted in FOXM1 up-regulation without prominent cell death induction. Whereas, the combination of tamoxifen and STL001 efficiently prevents tamoxifen-induced FOXM1 up-regulation and drastically enhances the cytotoxic effects of tamoxifen therapy, detected by induction of strong apoptosis indicated by caspase-3 cleavage (Fig. 6D) and loss of TAM-R cell viability (Fig. 6E). The results have confirmed that the combination of tamoxifen plus STL001 could restore the sensitivity to tamoxifen in tamoxifen therapy-resistant ER+ breast cancers.

Further, we have tested the sensitization effect of STL001 on ovarian cancer and triple-negative breast cancers (TNBC), which are very aggressive forms of malignancies in women. The aberrant over-expression of FOXM1 is the key molecular alteration in ovarian cancers [2] and TNBC [54] and is associated with chemotherapy resistance [2, 3]. Doxorubicin is one of the most commonly used anticancer drugs approved by the FDA for ovarian cancer, TNBC and various other types of malignancies. To test if STL001 can sensitize ovarian cancer (OVCAR-8, ES-2) cells and TNBC (HCC1143) to doxorubicin, OVCAR-8, ES-2 and HCC1143 cells were treated with sublethal concentrations of doxorubicin, display a significant increase in FOXM1 protein abundance, whereas the addition of STL001 in combination with doxorubicin efficiently prevented FOXM1 activation, resulting in decreased FOXM1 protein levels and potent induction of apoptotic cell death (indicated by caspase-3 cleavage) when compared with cells treated with doxorubicin chemotherapy alone (Fig. 6F, H). A similar sensitization effect was observed when HCC1143 cells were treated with STL001 in combination with cisplatin (Fig. 6I). However, the sensitization effect of STL001 was absent in ovarian cancer and TNBC cells with stable shRNA-mediated FOXM1-knockdown (Fig. 6G, J). These findings suggest that FOXM1 is a crucial factor in ovarian cancer and TNBC resistance to chemotherapy and mediates the effects of STL001 in the chemosensitization of cancer cells. In this study, we found that STL001 was effective in sensitizing a wide variety of cancer cells to a broad spectrum of anticancer drugs, through FOXM1 suppression, suggesting that FOXM1 is a crucial factor in therapeutic resistance in solid cancer and mediates the effects of STL001 in sensitization of solid cancer to different chemotherapies.

## Discussion

This study presents a novel FOXM1 inhibitor STL001 with similar biological properties to the parent compound STL427944 [5, 37]; however, STL001 is 25–50 times more effective in reducing the cellular FOXM1 activity in a variety of human cell lines derived from solid tumors (Fig. 1). The STL001 preserves the mode of action of the parent compound via cytoplasmic re-localization of FOXM1 and autophagic degradation [Fig. 2]. However, the exact mechanisms of the cytoplasmic re-localization of FOXM1 protein and autophagy induction by STL001 are currently under investigation. Of note, the mechanism of FOXM1 suppression by STL001 is completely different from other classes of FOXM1 inhibitors reported with less defined selectivity and a weak inhibition profile [38].

In the recent past, several small-molecule inhibitors of FOXM1 have been established, but none have advanced to clinical trials [38]. Currently, it is not clear whether targeting the FOXM1-DBD-DNA interactions is a promising strategy because FOXM1 can regulate transcription by forming complexes with several other TFs, which could limit the impact of this class of FOXM1 inhibitors [38]. Therefore, the development and testing of high-quality FOXM1 inhibitors is an important health need. We have previously described a first-in-class small-molecule FOXM1 inhibitor, STL427944 [5], identified by a completely different strategy, transcriptomic network analysis and confirmed as a selective inhibitor of FOXM1. In various cancer cells, STL427944 was efficiently blocking FOXM1 activity, providing a completely new mode of action; however, due to some metabolic liabilities, it worked at concentrations that are considered too high for a targeted inhibitor [5]. Considering its potential as a selective inhibitor of FOXM1, we wanted to improve it. The heterocyclic ring replacement in the parent compound STL427944 is likely to have significantly improved the overall stability observed in its derivative, STL001 (Fig. 1A). These structural modifications resulted in at least 25–50 times more active compound with greater drug-like properties and thus enhanced potency (Fig. 1B–G). The novel compound, STL001 has shown similar activity in AML [37] and preserved the autophagy-dependent mode of action (Fig. 2A–C) of the parental compound, STL427944 [5].

The gene regulation by STL001 studied in the greatest detail, showed substantial overlap with that of STL427944 [5] and with stable shRNA-mediated FOXM1-KD cells (Figs. 3, 4). Transcriptome-based Gene Ontology enrichment analysis in the category of biological process predicted mitotic cell division and DNA replication processes were majorly affected (displayed negative normalized enrichment scores) by STL001 besides the inhibition of FOXM1 activity (Fig. 3B, C). These activities are well known to be under FOXM1 regulation [38]. Thus, we can conclude that most gene expression changes caused by STL001 treatment are consequent of FOXM1 inhibition. In contrast, Gene Ontology enrichment analysis also highlighted steroid/cholesterol biosynthetic process and negative regulation of protein secretion with all the upregulated genes (Fig. 3B, C and Table 1). This is a completely new activity of FOXM1. Recently, FOXM1 has been shown to be involved in the mevalonate pathway of cholesterol biosynthesis, and inhibitors of the cholesterol biosynthetic pathway reduced FOXM1 expression [55]. It shows that cholesterol biosynthetic pathway components act as upstream FOXM1 regulators, but their regulation by FOXM1 indicates more complex relations, possibly a negative feedback loop between FOXM1 and cholesterol biosynthetic pathways.

Additionally, GSEA enrichments revealed the suppression of cell cycle and mitotic checkpoint regulatory (AURKB, PLK1, and E2F) and the DDR and DNA-repair (ATR, BARD1, and FANCONI) pathways by STL001 in esophageal cancer (Fig. 3D). Notably, DE genes by STL001 showed substantial overlap with the FOXM1-KD group in ovarian cancer (Fig. 4B, C). Moreover, GSEA revealed the suppression of FOXM1_pathway and AURORA_B_pathway as common pathways in both the STL001-treated and FOXM1-KD ovarian cancer cells (Fig. 4D). This verified the selectivity of STL001 towards the FOXM1 regulatory network. It is noteworthy that the negative effect of STL001 on FOXM1_Pathway and AURORA_B_Pathway shows its direct effect on FOXM1 activity. Of interest, AURKB and PLK1 are the well-known targets of FOXM1 [40], also the activity of E2F can be affected by FOXM1 [41]. While ATR, BARD1, and FANCONI are DDR pathways, several crucial gene components of these pathways are regulated by FOXM1 in DNA damage repair [10]. Thus, we propose that the impairment of these pathways by STL001-mediated FOXM1 suppression may contribute to the increased vulnerability of FOXM1-deficient cancer cells universally to a broad range of chemotherapeutic drugs. Based on these results, we conclude that the gene expression regulated by STL001 is mostly the consequent of the inhibition of FOXM1 activity in cancer cells, thereby supporting the idea that the novel FOXM1 inhibitor STL001 is highly selective in its activity toward FOXM1.

The sensitization of chemoresistant cancer cells to chemotherapies, especially to DNA-damaging agents, is the well-known effect of FOXM1 inhibition in cancer cells [5, 8, 20–24, 37, 38]. We expected that STL001, being a more effective FOXM1 inhibitor as compared to its parental compound STL427944, should sensitize solid tumors-derived cancer cell lines more efficiently to current chemotherapies. As anticipated, we found that STL001, which is not exerting prominent cytotoxic effects on its own (Supplementary Fig. 1B, C), sensitizes cancer cells to a broad spectrum of conventional chemotherapeutic drugs (5-fluorouracil, irinotecan, cisplatin, doxorubicin, paclitaxel, and tamoxifen) widely used for the treatment of solid cancer patients (Figs. 5, 6). This also proved FOXM1 as a crucial factor that evokes drug resistance more universally to a broad spectrum of current anticancer therapies. However, STL001-induced FOXM1 suppression can sensitize a wide variety of human solid cancers of different origin to apoptosis induced by anticancer drugs (Figs. 5, 6). The findings of this study on solid cancers are consistent with our recent report in AML [37], which confirms that STL001-induced FOXM1 suppression could be a good therapeutic strategy to overcome drug resistance in both solid tumors and hematological malignancies such as AML. The chemotherapy options for patients with the most aggressive types of cancers such as ovarian, colorectal, breast, prostate, and esophageal cancer remain very limited because of the acquired resistance to conventional chemotherapies [3, 5, 6, 10, 37, 38, 44]. In the present perspective, the combination of different chemotherapies with synergistic effects is considered a more efficient approach in modern cancer therapeutics [38, 43]. Combination therapies have great advantages, as it is a more efficient way to eradicate cancer with lower doses and thus less undesired adverse effects. Therefore, STL001 offers the potential for combination therapy in the clinic.

Given that the FOXM1 inhibitor STL001 is a novel agent, the exact details of its functional interactions with chemotherapy are unknown; there was a possibility that STL001 may have functional interactions with chemotherapeutic drugs through other mechanisms, independent of FOXM1. However, using stable shRNA-mediated FOXM1-KD cell lines, we have demonstrated that FOXM1-deficient cell lines, which were already sensitive to chemotherapies, could not be further sensitized by STL001 (Figs. 5I and 6B, G, J). It suggests that the sensitization effect of STL001 in different cancer cells is conveyed specifically through FOXM1 suppression.

The novel FOXM1 inhibitor STL001, developed by the first-generation modification in its parental compound STL427944, is more efficient in reducing the cellular FOXM1 activity in a variety of solid cancers and AML and potentially has the same high selectivity towards FOXM1. Notably, STL001 does not exert prominent cytotoxic effects on its own; however, it sensitizes cancer cells to a broad spectrum of conventional chemotherapeutic drugs most probably by increasing the vulnerability of cancer cells via suppressing the activity of FOXM1 and its downstream pathways involved in cancer survival and drug resistance. Further, in vivo investigations are required to evaluate the efficacy of FOXM1 inhibitor STL001 either alone or in combination with other agents. However, this novel compound offers intriguing translational opportunities for the development of new anticancer agents as combination therapies targeting FOXM1 activities in a variety of human cancers.

## Materials and methods

### Cell culture

The human distal oesophageal adenocarcinoma cell line (FLO-1; ECACC 11012001) was purchased from Sigma-Aldrich. C3-luc cell line expressing FOXM1-EGFP fusion protein controlled by doxycycline-inducible CMV promoter was derived from U2OS human osteosarcoma cells as described earlier [39]. LNCaP and 22Rv1 cell lines (human prostate carcinoma) were provided by Dr. D. J. Vander Griend (University of Illinois at Chicago, Chicago, IL, USA) and Dr. D. G. Tang (Roswell Park Cancer Institute, Buffalo, NY, USA). Human OVCAR-8 and ES-2 cell lines (High-grade serous ovarian cancer, HGSOC) were provided by Dr. J. Burdette (University of Illinois at Chicago). HCT-116 and HCT-FET cell lines (human colorectal carcinoma) were provided by Dr. B. Jung (University of Illinois at Chicago). Triple-negative breast cancer (TNBC) cell line, HCC-1143 was provided by Professor Debra Tonetti (University of Illinois at Chicago).TAM-R (Tamoxifen resistant MCF-7) human breast cancer cell line was provided by Dr. Hisham Mohammed (Oregon Health & Science University, Portland, USA). LNCaP and 22Rv1 cell lines were cultured in RPMI-1640 with 2.0 mM L-Glutamine (Gibco; Thermo Fisher Scientific, Waltham, MA, USA). HCC-1143 cell lines were cultured in Iscove’s Modified Dulbecco Medium (IMDM) with 2.0 mM L-Glutamine (Gibco; Thermo Fisher Scientific). FLO-1, C3-luc, OVCAR-8, ES-2, HCT-116, and HCT-FET cell lines were cultured in Dulbecco’s Modified Eagle Medium (DMEM) with 4.5 g/L glucose and 4 mM L-Glutamine (Gibco; Thermo Fisher Scientific). For all cell lines, the growth media was supplemented with 10% Foetal Bovine Serum (FBS), penicillin (100 U/mL), and 100 μg/mL streptomycin (Gibco; Thermo Fisher Scientific).TAMR cells were routinely cultured in DMEM/F12 medium without phenol red (Gibco; Thermo Fisher Scientific), containing 1% charcoal-striped FBS, 2.5 mM L-Glutamine (Thermo Fisher Scientific), 6.0 ng/mL insulin (Millipore Sigma) and 50.0 nM 4-hydroxytamoxifen (4-OHT; Millipore Sigma). All cell lines were grown and maintained at 37 °C in a humidified incubator with 5% CO_2_. Sub-confluent cultures (70–80%) were split 1:5 using 0.25% Trypsin/EDTA (Millipore Sigma).

Cells were confirmed to be mycoplasma-free by routine testing using PCR-based tests and DAPI-staining with subsequent evaluation by biological fluorescence microscopy.

### Chemical compounds and drugs

STL427944 and its derivative STL001 (Vitas-M Laboratory, Hong Kong), Paclitaxel (APExBIO Technology, USA), 5-FU (LKT Laboratories, USA), Doxorubicin (Thermo Fisher Scientific), and Irinotecan (Millipore Sigma) were dissolved in DMSO (Millipore Sigma). Tamoxifen (Millipore Sigma) was dissolved in ethanol. Cisplatin (AdipoGen Life Sciences, USA) was dissolved in 5% D-glucose solution in sterile water. Doxycycline (LKT Laboratories) and Puromycin (Millipore Sigma) were dissolved in sterile water. Estrogen, β-Estradiol (E2) was provided by Dr. Hisham Mohammed (Oregon Health & Science University) dissolved in ethanol.

### Drug treatment of cultured cells

After harvesting, cells were counted in the presence of Trypan Blue (Thermo Fisher Scientific) and seeded onto tissue culture plates to achieve ~50% confluency. The next day, treatment of cells was performed by aspirating the non-adherent cells and growth medium and replacing it with the fresh one containing selected concentrations of drugs. For Tamoxifen based studies, the non-adherent cells and growth media were aspirated and replaced with the DMEM supplemented with 4.5 g/L glucose and 4.0 mM L-Glutamine, 10% FBS, penicillin (100 U/mL), 100 μg/mL streptomycin, 10.0 nM estrogen, and selected concentrations of drugs. The vehicle control groups were treated using the solvent of the drug, vehicle concentration did not exceed 0.3%. Post-treatment at selected times, the cells were harvested, washed with ice-cold PBS and used for protein or RNA purification as described below.

### Stable FOXM1-expression knockdown in cancer cells

FLO-1, HCT-116, OVCAR-8, and HCC1143 cells were seeded on commercially available 12-well tissue culture plates to achieve ~40% confluency. The next day, cells were transduced with MISSION® lentiviral particles carrying pLKO.1 vector encoding a non-targeting shRNA control or shRNA against human FOXM1 transcripts (Millipore Sigma) at multiplicity of infection (MOI) 10 in the presence of Polybrene (10 μg/mL) and allowed to incubate for 24 h at 37 °C in a humidified incubator with 5% CO_2_. Transduced cells were selected by their cultivation with puromycin (1.0 μg/mL) for 10 days and then maintained without puromycin as described above.

### Protein immunoblotting

Total protein was extracted using ice-cold radio-immunoprecipitation assay (RIPA) buffer (Millipore Sigma) supplemented with Halt protease- and phosphatise-inhibitor cocktails (Fisher Scientific), 2.0 mM sodium orthovanadate (New England Biolabs, Inc., USA), and 5.0 mM sodium fluoride (Millipore Sigma) according to the manufacturer’s protocol. Protein content in each sample was estimated using Bio-Rad Protein Assay (Bio-Rad, USA). Equal amounts of protein (20–30 µg) were separated on hand-cast SDS/PAGE (6–12%) mini-protein gels and transferred to 0.2 µm Immobilon-Psq polyvinylidene difluoride (PVDF) transfer membrane (Millipore Sigma). Membranes were blocked with 4% bovine serum albumin (BSA; Millipore Sigma) in TRIS-buffered saline (TBS) with 0.1% Tween-20 (TBS-T, Thermo Fisher Scientific) and probed overnight at 4 °C with the primary antibodies (Supplementary Table 1) diluted in 5% BSA in TBS-T. When appropriate, the membranes were then washed with TBS-T for 15 min and proved with the HRP-conjugated secondary antibodies (Supplementary Table 1) for 2 h at room temperature. After incubating membranes with the secondary antibodies, the membranes were then washed three times for 10 min each with TBS-T. Protein bands were developed using SuperSignal™ West Pico PLUS Chemiluminescent Substrate (Thermo Fisher Scientific) and detected using ChemiDoc Imaging System (Bio-Rad). The molecular weights of protein makers are indicated on the right of each immunoblot image in the figures.

### Full-transcriptome RNA-seq

Total RNA from cultured cells was extracted and purified using TRIzol reagent (Fisher Scientific) and the PureLink™ RNA Mini Kit (Fisher Scientific) including on-column DNase (Thermo Fisher Scientific) treatment according to the manufacturer’s instructions. To assess the integrity of RNA, all samples were analyzed on the Agilent 4200 TapeStation (Agilent Technologies, USA). The remaining DNA concentrations were measured using the Qubit fluorometer (Thermo Fisher Scientific). In all the samples the DNA amounts did not exceed 2% of the total amount of nucleic acid.

Sequencing libraries for the Illumina sequencing platform were created in one batch in a 96-well plate, we used Stranded CORALL Total RNA-Seq Library Prep kit (Lexogen, Austria) with Lexogen’sRiboCop HMR rRNA Depletion Kit. In brief, in the first step during rRNA removal, we used 260–660 ngs of total RNA, and then followed by library creation initiated with random oligonucleotide primer hybridization with complementary sequence within the RNA template and reverse transcription. No prior RNA fragmentation was done before reverse transcription, as the insert size was determined by proprietary size-restricting method. Next, Illumina-compatible P5 sequences and UMIs (Unique Molecular Identifiers) were ligated at the 3′ end of the first-strand cDNA fragments. During the following steps of the 2nd-strand cDNA synthesis and the double-stranded cDNA amplification, unique i5 and i7 index sequences as well as complete adapter sequences required for cluster generation were added. The number of PCR amplification cycles was 12, as determined by qPCR using a small pre-amplification library aliquot for each sample.

Subsequently, the final PCR amplified libraries were purified and quantified. Finally, prior to sequencing average fragment sizes were confirmed to be 325 bp by Agilent 4200 Tape Station (Agilent Technologies). The final library pool concentration was confirmed by qPCR and then subjected to test sequencing in order to check sequencing efficiencies and adjust accordingly the proportions of individual libraries. Sequencing was carried out on the Illumina NovaSeq 6000 system with S4 flow cell (Illumina, USA), approximately 30 M 2 × 150-bp clusters per sample.

### Bioinformatical analysis of RNA-seq data

Sequencing data were aligned to human reference genome version GRCh38 annotated by Gencode version 43, using STAR [PMID: 23104886]. Counts within genes were obtained by Feature Counts [PMID: 24227677]. Differential expression in STL001 versus control and in FOXM1-KD versus control for autosomal protein-coding genes was assessed by the likelihood ratio test, based on the negative binomial distribution as implemented in DESeq2 [PMID: 25516281]. Nominal *P*-values were adjusted for multiple comparisons using the Benjamini and Hochberg approach [Benjamini Y, Hochberg Y. Controlling the false discovery rate: a practical and powerful approach to multiple testing. Journal of the Royal Statistical Society Series B (Methodological) 1995;57:289–300]. Significant genes were determined by adjusted *P*-value < 0.05 and fold changes lower than 0.5 or higher than 2.0. NIH DAVID [PMID: 12734009] was used for gene enrichment in Gene Ontology biological processes [PMID: 10802651]. Gene set enrichment analysis [PMID: 16199517] in Pathway Interaction Database (PID) collection of curated and peer-reviewed canonical pathway gene signatures used the Preranked algorithm with a number of permutations set to 1000. Pathways with FDR < 0.01 were considered significant.

### Statistical analysis

At least three independent biological replicates were used for all experiments describing cell treatment with drugs. For immunoblot experiments, the images shown in the paper represent the results that were consistent across several independent experiments. The statistical tests used in each experiment are described in the corresponding figure and table legends. Statistical significance was accepted with *p* < 0.05. Statistical analysis and the graphs were generated using Excel.

### Supplementary information

Suppl figure legends

Suppl fig 1

Suppl table 1

Suppl table 2

Suppl table 3

Suppl table 4

Original Data File

## Data Availability

Raw RNA-seq data on gene expression levels in FLO-1 and OVCAR-8 cells treated with STL001 and stable shRNA-mediated FOXM1-KD in OVCAR-8 are available from Gene Expression Omnibus (accession ID GSE261182). Processed RNA-seq data on gene expression levels in FLO-1 and OVCAR-8 cells treated with STL001 and stable shRNA-mediated FOXM1-KD in OVCAR-8 are included in this paper as Supplementary Table 2 (FLO-1 cells), Supplementary Table 3 (OVCAR-8 cells), and Supplementary Table 4 (OVCAR-8_FOXM1-KD).
