# Supplementary material for: Novel FOXM1 inhibitor STL001 sensitizes human cancers to a broad-spectrum of cancer therapies
Source: Cell Death Discov. 2024 May 2;10:211. doi: 10.1038/s41420-024-01929-0 (PMC11066125; doi:10.1038/s41420-024-01929-0)
Supplement: Supplementary file 1 — Suppl figure legends [file 41420_2024_1929_MOESM1_ESM.docx]

**Supplementary Figure and table legends:**

**Supplementary Figure 1. STL001 is more efficient than STL427944 in suppressing FOXM1 activity without exerting prominent cytotoxic effects on its own.**

**Supplementary Table 1. List of antibodies used in immunoblotting.**

**Supplementary Table 2. Transcriptome-wide gene expression changes by STL001 treatment in esophageal cancer (FLO-1) cells.**

**Supplementary Table 3. Transcriptome-wide gene expression changes by STL001 treatment in ovarian cancer (OVCAR-8) cells.**

**Supplementary Table 4. Transcriptome-wide gene expression changes by stable shRNA-mediated FOXM1-knockdown (KD) in ovarian cancer (OVCAR-8) cells.**
