# Supplementary material for: Novel FOXM1 inhibitor STL001 sensitizes human cancers to a broad-spectrum of cancer therapies
Source: Cell Death Discov. 2024 May 2;10:211. doi: 10.1038/s41420-024-01929-0 (PMC11066125; doi:10.1038/s41420-024-01929-0)

**Supplementary Figure 1. STL001 is more efficient than STL427944 in suppressing FOXM1 activity without exerting prominent cytotoxic effects on its own.** (A) Esophageal cancer cells (FLO-1) were treated with different concentrations of STL001 (5 and 10 µM) and STL427944 (25 and 50 µM) for 24hrs. Total protein samples obtained from treated cells were analyzed for FOXM1protein levels via immunoblotting, and β-actin was used as internal loading control (n = 3 for each group). (B) FLO-1 cells were treated with 1, 5, 10, or 25 µM concentrations of STL001 for 24hrs. Total protein samples were obtained from cells immediately after treatment and analyzed for FOXM1, cleaved caspase-3 levels via immunoblotting, and β-actin was used as internal loading control (n = 3 for each group). (C) The numbers of viable cells were counted in presence of Trypan Blue and normalized to control (0 µM) sample. The results shown are the mean ± SEM of three independent experiments performed in triplicate (n = 3). The STL001 is more efficient in reducing cellular FOXM1 protein levels as compared to its parental compound STL427944; notably, as a single agent, STL001 is not exerting prominent cytotoxic effects.


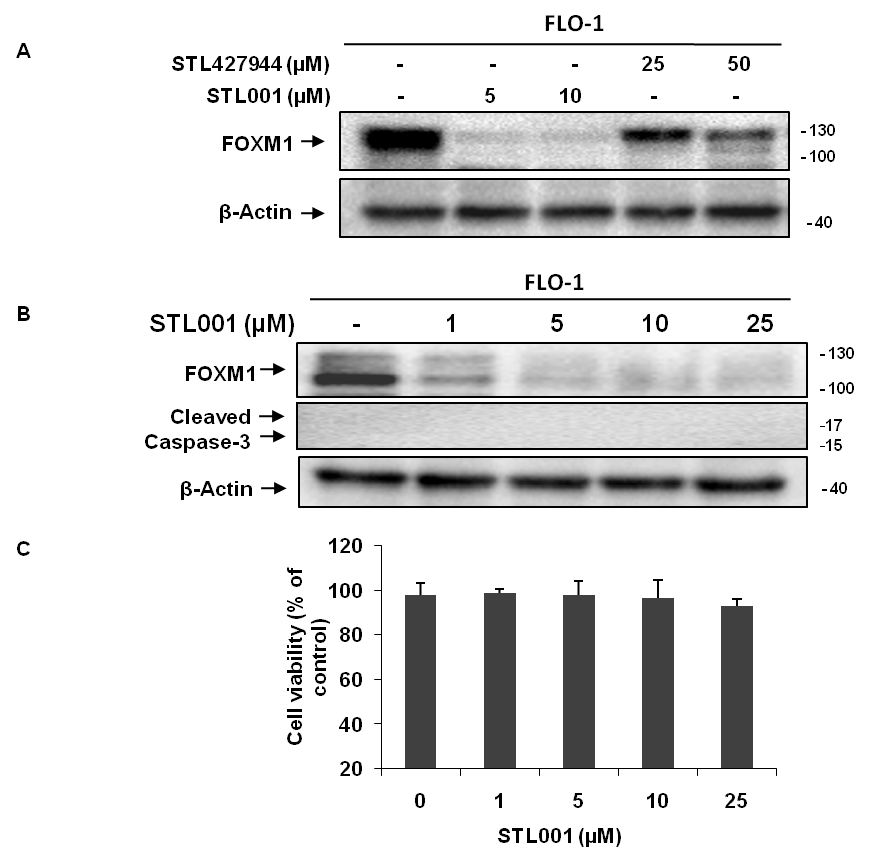

Supplement: Supplementary file 2 — Suppl fig 1 [file 41420_2024_1929_MOESM2_ESM.docx]
