# Supplementary material for: Novel FOXM1 inhibitor STL001 sensitizes human cancers to a broad-spectrum of cancer therapies
Source: Cell Death Discov. 2024 May 2;10:211. doi: 10.1038/s41420-024-01929-0 (PMC11066125; doi:10.1038/s41420-024-01929-0)
Supplement: Supplementary file 3 — Suppl table 1 [file 41420_2024_1929_MOESM3_ESM.docx]

**Supplementary table 1. List of antibodies used in immunoblotting.**

| **S.No.** | **Protein target** | **Supplier and cat. #** | **clone/ID and host** | **Working dilution** |
| --- | --- | --- | --- | --- |
| **1.** | FOXM1 | Cell Signaling Technology, USA; #5436 | D12D5; Rabbit mAb | 1:1,000 |
| **2.** | Cleaved Caspase-3 (Asp175) | Cell Signaling Technology, USA; #9664 | 5A1E; Rabbit mAb | 1:1,000 |
| **3.** | LC3A/B | Cell Signaling Technology, USA; #12741 | D3U4C; Rabbit mAb | 1:2,000 |
| **4.** | β-actin | Thermo Fisher, USA; # MA1-91399 | AC-15, Mouse mAb | 1:10,000 |
| **5.** | Anti-Rabbit IgG, HRP-conjugated | Jackson Immunoresearch, USA;  #611-035-215 | 611-035-215; Alpaca polyclonal | 1:10,000 |
| **6.** | Anti-Mouse IgG, HRP-conjugated | Jackson Immunoresearch, USA;  #715-035-150 | 715-035-150; Donkey polyclonal | 1:10,000 |
