# Supplementary material for: Novel FOXM1 inhibitor STL001 sensitizes human cancers to a broad-spectrum of cancer therapies
Source: Cell Death Discov. 2024 May 2;10:211. doi: 10.1038/s41420-024-01929-0 (PMC11066125; doi:10.1038/s41420-024-01929-0)

**Original Data.** Full uncropped images of blots shown in the paper (Figure 1C, 1D, 2A, 2C, 5I, 6B, 6G, and 6J). For each immunoblot image in the paper, molecular weights of protein markers are indicated on the right.


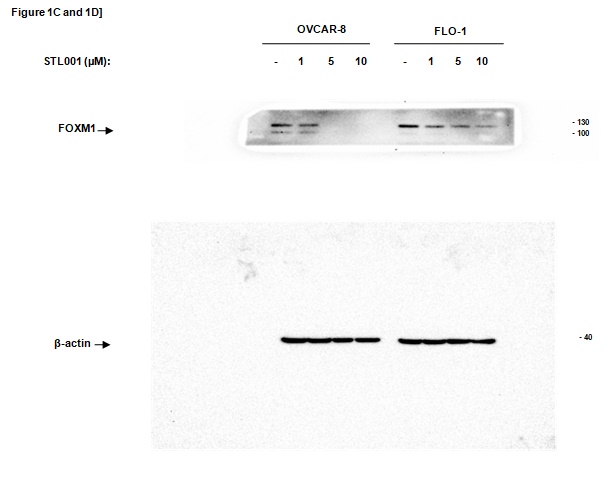


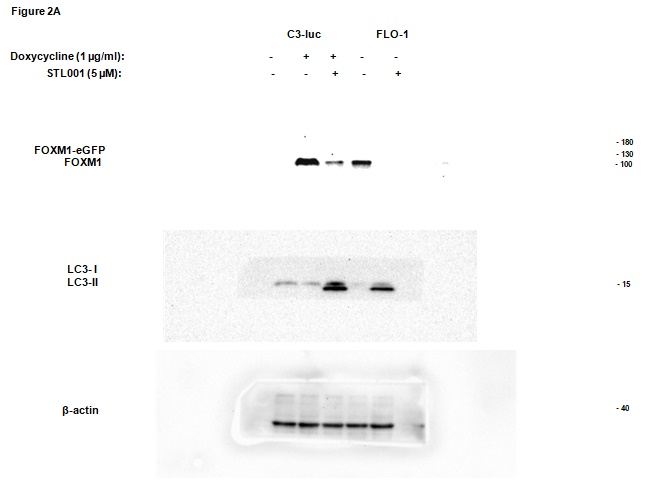


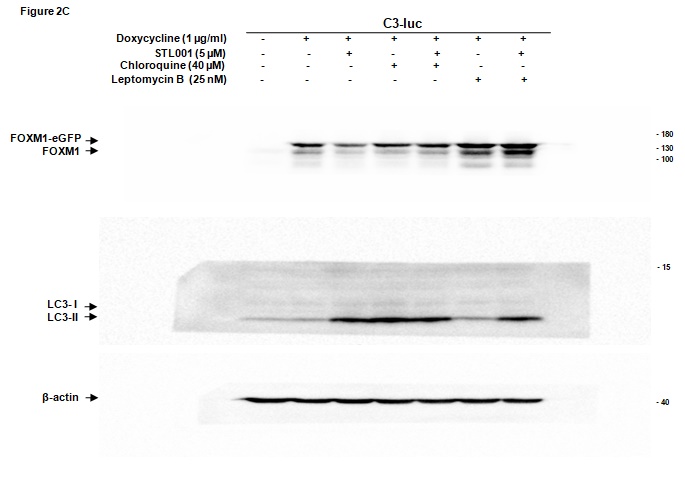


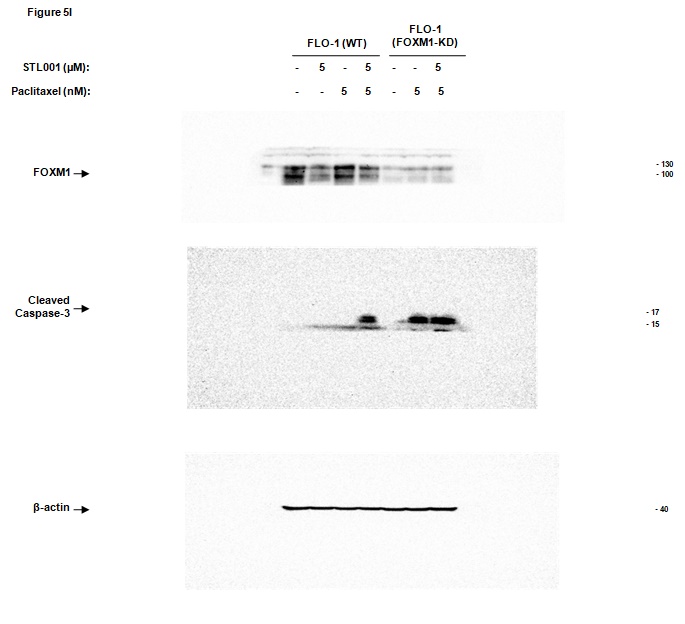


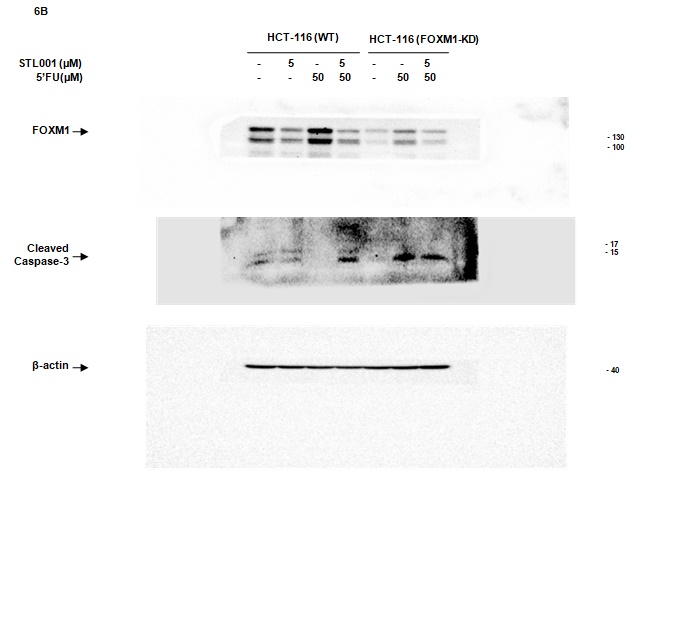


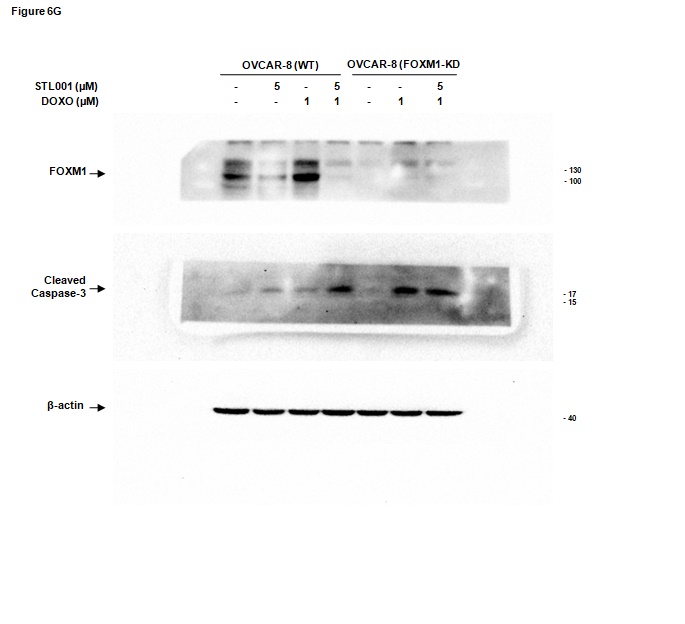


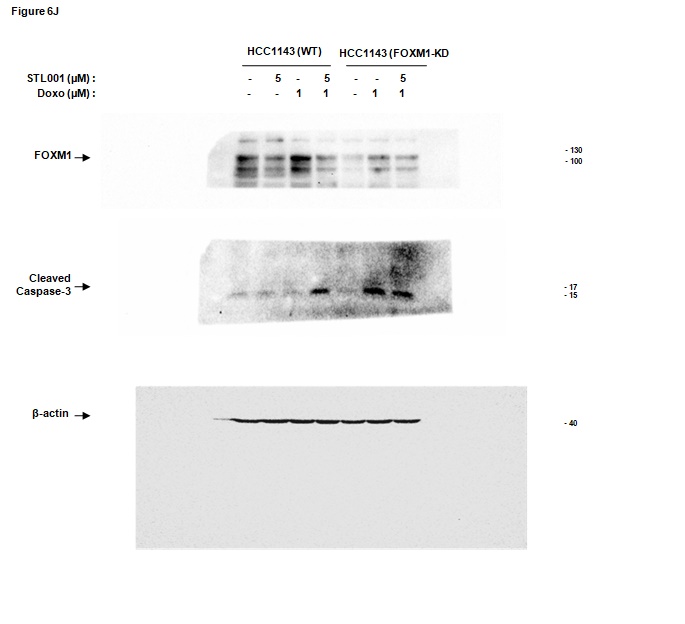

Supplement: Supplementary file 7 — Original Data File [file 41420_2024_1929_MOESM7_ESM.docx]
